# Anaerobic choline metabolism in microcompartments promotes growth and swarming of *P* *roteus mirabilis*

**DOI:** 10.1111/1462-2920.13059

**Published:** 2015-11-03

**Authors:** Eleanor Jameson, Tiantian Fu, Ian R. Brown, Konrad Paszkiewicz, Kevin J. Purdy, Stefanie Frank, Yin Chen

**Affiliations:** ^1^ School of Life SciencesUniversity of WarwickCoventryCV4 7ALUK; ^2^ School of BiosciencesUniversity of KentCanterburyKentCT2 7NJUK; ^3^ College of Life and Environmental SciencesUniversity of ExeterExeterEX4 4QDUK

## Abstract

*Gammaproteobacteria* are important gut microbes but only persist at low levels in the healthy gut. The ecology of *G* *ammaproteobacteria* in the gut environment is poorly understood. Here, we demonstrate that choline is an important growth substrate for representatives of *G* *ammaproteobacteria*. Using *P* *roteus mirabilis* as a model, we investigate the role of choline metabolism and demonstrate that the *cut* *C* gene, encoding a choline‐trimethylamine lyase, is essential for choline degradation to trimethylamine by targeted mutagenesis of *cut* *C* and subsequent complementation experiments. *P* *roteus mirabilis* can rapidly utilize choline to enhance growth rate and cell yield in broth culture. Importantly, choline also enhances swarming‐associated colony expansion of *P* *. mirabilis* under anaerobic conditions on a solid surface. Comparative transcriptomics demonstrated that choline not only induces choline‐trimethylamine lyase but also genes encoding shell proteins for the formation of bacterial microcompartments. Subsequent analyses by transmission electron microscopy confirmed the presence of such novel microcompartments in cells cultivated in liquid broth and hyper‐flagellated swarmer cells from solid medium. Together, our study reveals choline metabolism as an adaptation strategy for *P* *. mirabilis* and contributes to better understand the ecology of this bacterium in health and disease.

## Introduction

*Bacteroidetes*, *Firmicutes* and *Proteobacteria* are the dominant microbes in bacterial communities of the human gut, with the former two accounting for > 90% of microbial biomass in a healthy gut (Arumugam *et al*., 2011; Yatsunenko *et al*., 2012). *Bacteroidetes* and *Firmicutes* are generally strict anaerobes fermenting dietary and host‐derived proteins and carbohydrates, whereas many *Proteobacteria*, particularly those *Gammaproteobacteria* of the *Enterobacteriaceae* family are facultative anaerobes, which live in close proximity to the mucosa of the gut lumen (Eckburg *et al*., 2005; El Kaoutari *et al*., 2013; Winter *et al*., 2013b). An imbalance in gut microbiota, known as dysbiosis, is usually associated with a sudden increase in the abundance of facultative anaerobic *Gammaproteobacteria*, particularly *Enterobacteriaceae*, a characteristic of gut malfunction and intestinal inflammation (Winter *et al*., 2013b). Understanding the mechanisms underpinning the sudden increase of *Gammaproteobacteria* during gut microbiota dysbiosis is essential to gain better knowledge of the association between gut dysbiosis and a number of bowel diseases, e.g*.* Crohn's disease (Baumgart *et al*., 2007), irritable bowel syndrome (Krogius‐Kurikka *et al*., 2009) and necrotizing enterocolitis (Normann *et al*., 2013).

A growing body of evidence has emerged over the past decade which supports the interplay between the metabolism of gut microbiota and the host response leading to an increase in facultative *Gammaproteobacteria* during gut dysbiosis (reviewed in Winter *et al*., 2013a). For example, many *Gammaproteobacteria* can utilize tetrathionate as an alternative electron acceptor (Winter *et al*., 2010). Tetrathionate is produced during gut inflammation by the oxidation of hydrogen sulfide, a metabolite abundant in a normal gut through the action of fermentation by *Firmicutes* and *Bacteroidetes* with subsequent reduction of sulfate by sulfate‐reducing bacteria (Winter *et al*., 2010). Gut inflammation also induces the formation of a number of other electron acceptors such as trimethylamine *N*‐oxide (TMAO) and nitrate, which can support anaerobic respiration of these facultative *Gammaproteobacteria* but not obligate anaerobic *Firmicutes* or *Bacteroidetes* (Winter *et al*., 2013b). Nitrate is generated through the oxidation of reactive nitrogen species released from neutrophils (Szabo *et al*., 2007), and TMAO was postulated to be formed from the oxidation of trimethylamine (TMA) as a result of gut microbiota metabolism (Winter *et al*., 2013b).

Not only can *Gammaproteobacteria* utilize these alternative host‐derived electron acceptors, but they can also acquire carbon and energy from host‐derived molecules. For example, *Salmonella* spp. can utilize ethanolamine anaerobically as a carbon and energy source. Ethanolamine is derived from the phospholipid phosphatidylethanolamine, a constituent of the membranes of gut epithelial cells, which is constantly produced during luminal tissue sloughing (Price‐Carter *et al*., 2001; Thiennimitr *et al*., 2011). Metabolism of ethanolamine by *Salmonella* is carried out in a unique bacterial organelle, namely a bacterial microcompartment (Chen *et al*., 1994; Penrod and Roth, 2006). It is thought that these microbial organelles optimize pathways with toxic or volatile intermediates (Havemann *et al*., 2002; Penrod and Roth, 2006). The protein shell of the ethanolamine utilization (Eut) microcompartments acts as a selective barrier that helps to retain volatile aldehyde intermediates and channel intermediates to the next pathway enzyme by colocalization of critical enzymes (Chowdhury *et al*., 2014).

Another major phospholipid in the gut epithelium is choline‐containing phosphatidylcholine (Kawai *et al*., 1974). It is known that choline can be a precursor for TMA and the enzyme catalyses this reaction, CutC, has recently been identified as a glycyl radical‐containing protein (Craciun and Balskus, 2012). Interestingly, the *cutC* gene cluster also contains a set of proteins that are likely to form microcompartments (Craciun and Balskus, 2012). Although the *cut* gene cluster was originally characterized from a sulfate reducer, *Desulfovibrio desulfuricans* (Craciun and Balskus, 2012), subsequent analyses have shown that *cutC* homologues and the shell proteins involved in microcompartment formation also occurred in some gut *Gammaproteobacteria* (Axen *et al*., 2014). Indeed, a very recent study has shown that these *cut*‐containing *Gammaproteobacteria*, including *Proteus mirabilis*, can form TMA from choline (Martínez‐del Campo *et al*., 2015). Therefore, this leads to the question – what are the benefits for choline metabolism in these *Gammaproteobacteria*?

In this study, we use the gram‐negative enteric *P. mirabilis*, a bacterium capable of colonizing the gut and the urogenital tract (Burall *et al*., 2004), as a model to investigate the role of choline metabolism. We demonstrated that *cutC* is essential in choline degradation to TMA in this bacterium by mutagenesis and subsequent complementation analyses. We provide the first evidence of a novel choline‐metabolizing microcompartment that is present in both vegetative and swarming cells. We show that choline degradation to TMA promotes anaerobic growth of this bacterium in liquid culture. Furthermore, we demonstrate, for the first time, true anaerobic swarming behaviour of this bacterium and reveal that choline degradation promotes the swarming‐associated colony expansion rates on solid agar surface. Together, our study contributes a potential explanation to the success and competitiveness of the *cut*‐containing *P. mirabilis* in gut dysbiosis (Garrett *et al*., 2010).

## Results

### Identification of a functional choline‐TMA lyase in *P* *. mirabilis*

**Figure 1:**
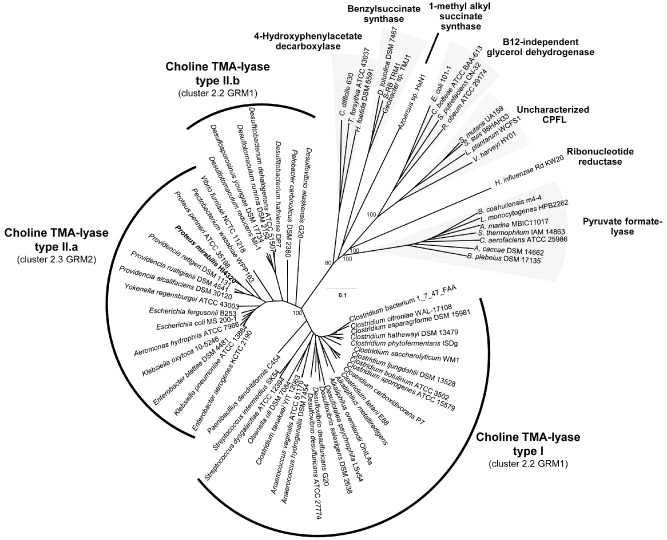
Neighbour‐joining phylogenetic tree, constructed from amino acid sequences of glycyl radical enzymes. The choline trimethylamine‐lyase, CutC, has been further split into two distinct sub‐clusters, type I and type II as previously described by Martínez‐del Campo and colleagues (2015). Association of the CutC clusters with different glycyl radical enzyme‐containing microcompartment (GRM) loci is given in bracketed bales, as defined by Axen and colleagues (2014). The *cut* *C* genes corresponding to type I and II.b sub‐clusters are approximately 2.5 kb and include the functionally characterized *cut* *C* of *D* *. desulfuricans.* Type II.a genes include the *cut* *C* of *P* *. mirabilis* and are approximately 3.4 kb in length. The other sequence clusters represent characterized glycyl radical enzymes, except the cluster labelled ‘Unknown CPFL’ which has an unknown function (Lehtiö and Goldman, 2004). Bootstrap values > 70% are indicated on the nodes (1000 replicates). The scale bar indicates evolutionary distance in mutations per residue.

The enzyme responsible for the conversion of choline to TMA has recently been identified from *Desulfovibrio desulfuricans* (Craciun and Balskus, 2012). This enzyme belongs to the glycyl radical enzyme family. We have analysed the phylogeny of CutC, a choline‐TMA lyase, in genome‐sequenced bacteria (Fig. 1). The resultant phylogenetic analysis shows two distinct clusters of CutC proteins, as previously reported by Martínez‐del Campo and colleagues (2015). The type I cluster is primarily comprised of obligate anaerobes including the characterized CutC from *D. desulfuricans* (Craciun and Balskus, 2012), whereas the type II cluster contains facultative anaerobes, including *P. mirabilis* and many isolates of the *Enterobacteriaceae* family. The type II CutC cluster is further split in to II.a containing *Gammaproteobacteria* and II.b containing *Firmicutes* and *Deltaproteobacteria*. A striking difference of these *cutC* genes appears to be in their length: type I and II.b *cutC* are approximately 2.5 kb in length, while type II.a are longer, with approximately 3.4 kb. The type II.a CutC enzymes have an elongated N‐terminus compared with those in cluster I and II.b, while the rest of the sequence is highly conserved (see sequence alignment in Fig. S1).

**Figure 2:**
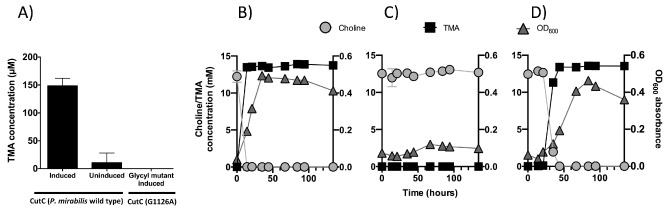
A. Quantification of trimethylamine (TMA) produced from recombinant *E* *. coli* carrying the codon‐optimized *P* *. mirabilis cut* *D* gene and either wild‐type *cut* *C* (first two bars) or a glycyl radical site CutC mutant (G1126A, third bar) during incubations in the defined medium supplemented with 1 mM choline chloride. Error bars represent standard deviations from three replicate cultures. B–D. Choline degradation and TMA production overlaid with growth in anaerobic *P. mirabilis* culture. B. *P* *roteus mirabilis* wild‐type, C. *P* *roteus mirabilis cut* *C* *::kan* mutant and D. *P* *roteus mirabilis cut* *C* *::kan* mutant complemented with native *cut* *CD*.

We then performed a heterologous expression experiment in *Escherichia coli* to validate whether *P. mirabilis* CutC and the accompanying activating enzyme CutD were sufficient for TMA formation from choline. We cloned *cutCD* into *E. coli* under an inducible T7‐*lac* promoter and monitored anaerobic production of TMA from choline. This resulted in inducible TMA production (Fig. 2A). The addition of the high concentration of salt to the M9 medium, to increase expression of the native *E. coli* high‐affinity choline transporter, *betT*, (Lamark *et al*., 1991) also had the added benefit of increasing the expression of *cutC*, which resulted in a visible protein band of the full length (126 kDa) on the SDS‐PAGE gel (Fig. S2), the identity of which was further confirmed by peptide sequencing using matrix‐assisted laser desorption/ionization – time of flight (MALDI‐TOF) mass spectrometry. Both the *cutC* and *cutD* genes were required to form TMA from choline since *E. coli* expressing either *cutC* or *cutD* alone was unable to degrade choline to TMA (data not shown). Site‐directed mutagenesis of the *cutC* gene expressed in *E. coli* confirmed that the glycine residue at the predicted glycyl radical site (G1126) is essential in catalysis and TMA production was completely abolished in the glycyl radical site mutant (Fig. 2A). Overall, our data confirm that the *cutC* homologue in *P. mirabilis* encodes a functional choline‐TMA lyase, although phylogenetic analyses clearly separate it from the only characterized CutC of *D. desulfuricans* (Craciun and Balskus, 2012).

To verify that the *cutC* gene was solely responsible for choline degradation in *P. mirabilis*, the *cutC* gene was inactivated by the insertion of a kanamycin gene cassette. Growth experiments confirmed that the *P. mirabilis cutC::kan* mutant no longer produced TMA and choline remained unused in the culture medium (Fig. 2C). However, when the mutant was complemented with the native copy of the *cutCD*, conversion of choline to TMA was restored (Fig. 2D). Together, these experiments demonstrated that CutC and CutD are responsible for anaerobic choline degradation to TMA in *P. mirabilis*.

### Choline metabolism enhances anaerobic growth of *P* *. mirabilis* in liquid medium

**Figure 3:**
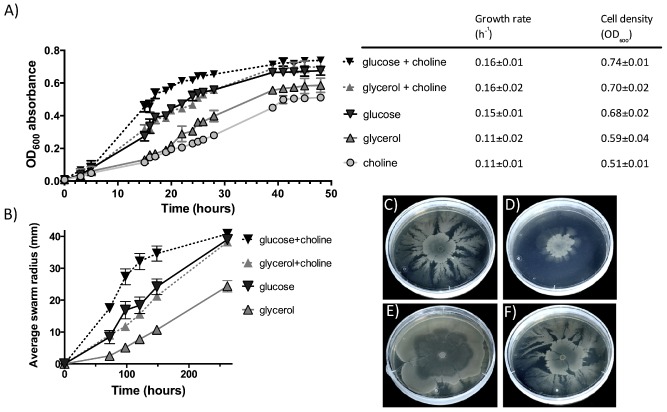
A. Anaerobic growth of *P* *. mirabilis* in liquid broth cultures in a defined medium at 37°C. The available carbon sources for *P* *. mirabilis* are indicated in the legend: glucose and choline (dashed 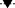), glucose only (solid 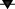), glycerol and choline (dashed 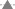), glycerol only (solid 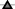) and choline only (solid 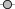). Error bars represent standard deviation for three replicate cultures. B. Cumulative anaerobic swarm radiuses of *P* *. mirabilis*, incubated at 30°C, inoculated from an aerobic stationary phase culture. Error bars show standard deviation for three replicate plates. The carbon sources on the swarming agar plates are glucose and choline (dashed 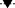), glucose only (solid 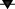), glycerol and choline (dashed 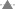) or glycerol only (solid 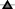). Maximum swarm radius is 42 mm on petri dishes. C–F. Anaerobic swarming pattern of *P* *. mirabilis* on agar plates supplemented with (C) glycerol and choline (10 days growth), (D) glycerol (10 days growth), (E) glucose and choline (6 days growth) and (F) glucose (10 days growth).

We next investigated the role of choline metabolism in *P. mirabilis*. Broth cultures of *P. mirabilis* were grown under fully anaerobic conditions in the defined medium supplemented with choline and sodium fumarate as an electron acceptor. A comparison was made between three different carbon sources; glucose, glycerol or choline. The optical density at 600 nm (OD_600_) was measured over a time course. All cultures grew, confirming that *P. mirabilis* was able to use choline as a sole carbon source for growth (Fig. 3A). Of the single carbon sources tested, the cultures with glucose alone grew fastest and reached the highest OD, followed by glycerol alone and then choline alone. Combining choline with either glucose or glycerol resulted in increased growth rates and increased final cell yields. The final OD_600_ for these two cultures were not significantly different and the culture grown on choline alone did not enter a logarithmic growth phase during the experiment.

### Choline metabolism promotes anaerobic swarming of *P* *. mirabilis*

The *P. mirabilis* culture was able to swarm both aerobically and anaerobically on minimal media. The growth phase and source of the *P. mirabilis* inoculum (i.e. from liquid broth or swarm agar plates) was vital to swarming‐associated colony expansion rates. Colony expansion occurred at the fastest rate when established anaerobic swarm cells were used to re‐inoculate a fresh plate, and slower swarming expansion rates were observed when liquid or aerobic cultures were used as the inoculum (data not shown). Anaerobic swarming‐associated growth on low nutrient medium did not result in a uniform ring but formed a dendritic growth pattern from irregular points around the initial inoculation colony (Fig. 3C–F). The consolidation rings demarking shorter swimmer cells between swarming intervals were not well defined, compared with aerobic swarming patterns on rich medium (for review see Williams and Schwarzhoff, 1978).

**Figure 4:**
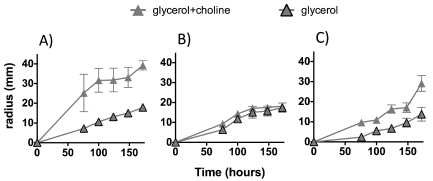
Cumulative anaerobic swarming radiuses of *P* *. mirabilis*. Error bars show standard deviation for three replicate plates, grown on glycerol or glycerol plus choline for (A) wild‐type, (B) *cut* *C* *::kan* mutant and (C) *cut* *C* *::kan* mutant complemented with native *cut* *CD*.

Different rates of swarming‐associated colony expansion were observed for *P. mirabilis* grown on glucose or glycerol as a sole carbon source and glucose or glycerol plates supplemented with additional choline (Fig. 3B). Colony expansion was fastest on glucose, while glycerol showed the slowest swarm rate. The addition of choline to glucose and glycerol plates increased colony expansion speed and the uniformity of the colony formed. Increasing the choline concentration had a cumulative affect on colony expansion rate (Fig. S3A). The *P. mirabilis cutC::kan* mutant was able to swarm on glycerol agar plates (Fig. 4), however, unlike the wild‐type strain, the mutant showed no increase in colony expansion rates when the plates were supplemented with additional choline (Fig. 4). The complemented mutant, however, showed a modest increase of colony expansion rates in the presence of additional choline compared with the *cutC::kan* mutant, confirming the role of choline metabolism in promoting swarming in *P. mirabilis*. To verify that the mutant and complemented mutant did not suffer from defective growth, they were grown in liquid media with a carbon source of either glycerol, glycerol plus choline or choline alone, and the growths were compared with the wild type. The resultant data demonstrated no increased growth of the *cutC::kan* mutant on glycerol supplemented with choline (Fig. S4), therefore supporting the observation that addition of choline did not further enhance colony expansion rates of the *cutC::kan* mutant (Fig. 4).

### Choline induces microcompartment formation in liquid cultures and in swarming cells

Transcriptomic analysis of *P. mirabilis* cultivated on glucose with/without additional choline indicates that the most highly differentially expressed genes code for proteins of the predicted choline utilization (*cut*) operon, including *cutC* and *cutD* and several genes annotated as microcompartment shell proteins, e.g. PMI2722, PMI2721, PMI2720, PMI2718 and PMI2714 (Table 1). The organization of the *P. mirabilis* genetic *cut* island and the proposed enzyme functions are outlined in Fig. S5. PMI2720, PMI2721, PMI2722, PMI2714 are homologues to the shell proteins PduJ/PduA in the 1,2‐propanediol utilization (Pdu) microcompartments and EutM in the ethanolamine utilization (Eut) compartment (Kofoid *et al*., 1999; Crowley *et al*., 2010), whereas PMI2718 shares sequence similarity with the EutN/CcmL family of shell proteins (Kofoid *et al*., 1999) (sequence similarity search at http://www.genome.jp/tools/blast/). The transcriptomic data suggested that choline may induce the production of shell proteins and hence the formation of microcompartments in *P. mirabilis* as previously reported for the related *cut* clusters of *D. desulfuricans* and *Desulfovibrio alaskensis* (Kuehl *et al*., 2014; Martínez‐del Campo *et al*., 2015).

**Table 1:** Top 20 differentially expressed genes. Positive log_2_‐fold change indicates downregulation with the addition of choline and negative values indicate upregulation with choline

Locus tag	Gene name	Log_2_‐fold change	*P*‐value	Gene description
PMI2721	*eutM/pduA/J* homologue	−10.853338	8.58E‐38	Microcompartment protein (BMC domain, PF00936.14)
PMI2720	*eutM/pduA/J* homologue	−9.9694882	1.25E‐36	Microcompartment protein (BMC domain, PF00936.14)
PMI2722	*eutM/pduA/J* homologue	−9.3293139	2.06E‐27	Microcompartment protein (BMC domain, PF00936.14)
PMI2716	*cutC*	−7.3519216	3.39E‐26	Choline‐trimethylamine lyase, CutC
PMI2719	*cutF*	−7.9456261	1.66E‐20	Aldehyde dehydrogenase, CutF
PMI2715	*cutD*	−6.0539258	8.64E‐20	Choline‐trimethylamine lyase, activating enzyme, CutD
PMI2714	*eutM/pduA/J* homolog	−5.2834574	7.49E‐17	Microcompartment protein (BMC domain, PF00936.14)
PMI2718	*eutN*/*ccmL* homologue	−10.804654	1.71E‐15	Microcompartment/carboxysome/ethanolamine utilization protein (EutN_CcmL, PF03319.8)
PMI2711	*emrE* homologue	−8.6639517	7.24E‐13	Quaternary ammonium compound resistance protein
PMI2717	*cutO*	−9.6507023	1.08E‐10	Alcohol dehydrogenase
PMI2710	*emrE* homologue	−9.2090116	2.81E‐06	Multi‐drug resistance protein, EmrE homologue
PMI0695	*trxB*	−1.9182025	0.000123	Thioredoxin reductase
PMI2713	*cutH*	−5.5647202	0.000259	Phosphate acetyltransferase
PMI0501		−1.8393886	0.000698	Uncharacterized conserved protein YjdB, phage protein
PMIt054		3.6411425	0.001508	tRNA‐Met
PMI1739		−1.6050110	0.001628	Hypothetical protein YfaZ precursor, putative exporter protein
PMI3109	*fdoH*	−1.6200982	0.001691	Formate dehydrogenase‐O subunit beta, transmembrane
PMI0297		−1.7078902	0.001728	Fimbrial subunit
PMI1288	*ydfG*	−1.7860287	0.001896	NADP‐dependent L‐serine/L‐allo‐threonine dehydrogenase
PMI1955	*eco*	−1.6974899	0.002404	Protease inhibitor ecotin; homodimeric protease inhibitor

BMC, bacterial microcompartment; NADP, nicotinamide adenine dinucleotide phosphate.

**Figure 5:**
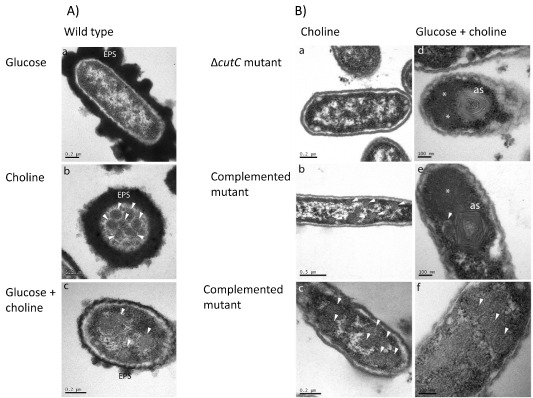
A. Transmission electron microscopy (TEM) micrographs of *P* *. mirabilis* wild‐type cultivated anaerobically in a defined medium supplemented with (a) glucose, (b) choline or (c) glucose plus choline as the sole carbon sources. B. Transmission electron microscopy micrographs of *cut* *C* *::kan* mutant (a, d) and the complemented mutant (b, c, e, f) cultivated anaerobically on choline or glucose plus choline as the sole carbon sources. Microcompartments are indicated by white arrows. EPS, extracellular polysaccharides; *indicates protein aggregation; as indicates aberrant microcompartment structures.

To determine if microcompartment formation is induced by growth on choline, *P. mirabilis* was cultured anaerobically in a liquid medium supplied with glucose, choline or glucose plus choline as sole carbon sources (Fig. 5). Samples were collected at regular time intervals (at 2 h, 4 h, 6 h, 8 h, 13 h, 25 h, 48 h, 77 h) for transmission electron microscopy (TEM) analysis of sections through the cells and to determine growth rate and TMA production (Figs S6–S7). Transmission electron microscopy imaging showed that in the presence of choline, microcompartments were observed from 6 h onwards. The time of appearance of the microcompartments coincided with an increase in OD and the production of TMA in cultures supplemented with choline. The OD began to increase at 4–6 h, and TMA was first detected at 4 h in cultures supplemented with choline (Fig. S7). In sections of *P. mirabilis* cultured solely on choline, the microcompartments appear better defined and easier to image than those grown on glucose plus choline (Fig. 5A). Nevertheless, they were detectable, whereas no microcompartments were observed in cells cultivated in the absence of choline.

**Figure 6:**
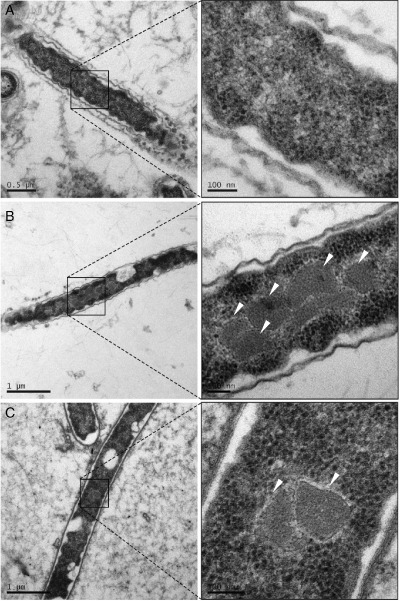
Transmission electron microscopy micrographs of *P* *. mirabilis* swarming cells. The swarming agar was supplemented with (A) glycerol, (B) choline or (C) glycerol plus choline as the sole carbon sources. Microcompartments are indicated by white arrows.

Microcompartment formation was severely disrupted in *P. mirabilis cutC::kan* mutant cultures (Fig. 5B). When transferring the *cutC::kan* cells from a starter culture containing glucose into choline supplemented minimal medium, the cells did not grow, and no microcompartment structures were detected by TEM. Whereas when the mutant strain was cultured on glucose and choline, aberrant multi‐layered structures and protein aggregations were observed (Fig. 5B). Complementation of the *cutC::kan* mutant with *cutCD* from a plasmid rescued the wild‐type microcompartment phenotype when grown on choline, while the complemented mutant cultured on choline plus glucose produced a combination of mal‐formed multi‐layered structures, protein aggregates and wild‐type microcompartments (Fig. 5B). Moreover, as choline has also been found to enhance anaerobic swarming of *P. mirabilis*, we prepared sections from anaerobic swarming plates and showed microcompartments were indeed present in elongated, hyper‐flagellated *P. mirabilis* swarmer cells, again only in the presence of choline (Fig. 6). Together, the simultaneous appearance of microcompartments in the complemented mutants with the onset of TMA production and cell growth demonstrates that these microcompartment structures are associated with choline metabolism.

## Discussion

Gut microbiota play important roles in our body, for example, in nutrient acquisition, development of mucosal immunity, metabolism of xenobiotics and the control of pathogens (Swann *et al*., 2009; Petersson *et al*., 2011; Reinhardt *et al*., 2012; Lawley and Walker, 2013). It has been well documented that *Bacteroidetes* and *Firmicutes* account for the majority of the microbiota in the healthy gut, while *Proteobacteria* only persist at low levels (Arumugam *et al*., 2011; Yatsunenko *et al*., 2012). *Proteobacteria* have been found to survive in close proximity to the gut mucosa (El Kaoutari *et al*., 2013; Winter *et al*., 2013b), an important source of phosphatidylcholine and its derivative choline (Kawai *et al*., 1974). It is known that gut microbiota convert quaternary amines (such as choline and carnitine) to TMA (Craciun and Balskus, 2012; Zhu *et al*., 2014). The mechanism for choline degradation to TMA in these *Gammaproteobacteria* combined with anaerobic respiration would allow them to take advantage of the host inflammatory response. Winter and colleagues (2013a) presented a potential mechanism for *Gammaproteobacteria* to exploit the host immune response to enhance anaerobic respiration. The host response limits the rich carbon sources in the gut contents through diarrhoea; however, host enterocytes and the gut mucosa provide a source of choline for *Gammaproteobacteria*, and the resultant TMA is able to react with host‐produced superoxide species to produce TMAO, fuelling anaerobic respiration (Balagam and Richardson, 2008). This mechanism of hijacking the host immune response provides an advantage to *Gammaproteobacteria* in a highly competitive environment. We have combined phylogenetic, metabolic and genetic analyses to confirm the presence and function of a choline utilization (*cut*) cluster in a group of human *Gammaproteobacteria*. It has long been reported that the gammaproteobacterial *P. mirabilis* (now shown to contain the *cut* cluster) can generate TMA when grown on choline (Dyer and Wood, 1947; Sandhu and Chase, 1986; Martínez‐del Campo *et al*., 2015). Using this facultative anaerobe, *P. mirabilis*, as a model we demonstrate that the Cut metabolic pathway increases growth rate and swarming colony expansion rate of cells *in vitro*.

We have observed that *P. mirabilis* can very rapidly uptake and degrade high concentrations of choline to TMA. The *cut* promoter is upregulated twofold in the presence of choline in liquid cultures (Fig. S8), and transcription of the *cut* gene cluster (PMI2710‐22) is strongly upregulated in the presence of choline (Table 1) as observed in *D. desulfuricans* (Martínez‐del Campo *et al*., 2015). The addition of choline leads to increased anaerobic growth rates in liquid culture. *Proteus mirabilis* can use choline as a carbon source but is unable to utilize the nitrogen and therefore excretes TMA. The anaerobic swarming of *P. mirabilis* on glucose was also enhanced by the addition of choline to a greater extent than that observed on glycerol. Choline was observed to promote swarming, increase the interval between consolidation phases and increase total colony expansion distance under anaerobic conditions (Fig. 3). We also present the first data on true anaerobic swarming by using minimal agar supplemented with fumarate (Fig. 3), whereas previously only swarming under aerobic conditions using the anaerobic respiratory chain with the aid of sodium azide has been demonstrated (Wilkerson and Niederhoffer, 1995; Alteri *et al*., 2012). Anaerobic swarming is important in the largely anaerobic human gut and urinary tract environments where *P. mirabilis* causes infection and has been implicated in conferring a competitive advantage (Armbruster and Mobley, 2012). The mechanism by which choline promotes swarming in *P. mirabilis* is, however, not entirely clear. It is known that numerous compounds (known as environmental cues, e.g*.* glutamine) can increase swarming activity even though they have no effect on cell growth rate (Armbruster *et al*., 2013). We postulate that the role of choline in swarming is likely linked to adenosine triphosphate (ATP) formation from the metabolites of downstream choline metabolism, such as acetyl‐CoA (Fig. S5). Comparison of swarming colony expansion rates on glycerol alone and glycerol with TMA indicates that the observed differences are due to choline metabolism and not the production of TMA (Fig. S3B). Our results show that the *cutC::kan* mutant had no enhancement of swarming in the presence of choline (Fig. 4), making the role of chemosensing in promoting swarming unlikely (Armbruster *et al*., 2013).

All the identified Cut clusters contain homologues of microcompartment proteins, suggesting that microcompartments play an important role in choline degradation (Fig. 1, Martínez‐del Campo *et al*., 2015). Indeed, in *D. alaskensis*, a mutant of a microcompartment gene was unable to utilize choline (Kuehl *et al*., 2014). The two types of *cutC* genes identified by Martínez‐del Campo and colleagues (2015) and Fig. 1 are, however, associated with different microcompartment genes (Axen *et al*., 2014). The *P. mirabilis cut* cluster belongs to the glycyl radical enzyme‐containing microcompartment (GRM2, cluster 2.3), which is predominantly found in human pathogens colonizing the gut or the urogenital tract (Axen *et al*., 2014). Five genes in the *P. mirabilis cut* operon are predicted to code for microcompartment shell proteins (PMI2722, PMI2721, PMI2720, PMI2718 and PMI2714). Through extensive TEM image analyses, we have confirmed that *P. mirabilis* cultures grown on choline indeed contained microcompartments (Figs 5, 6). We were also able to show that swarmer cells produce microcompartments, a surprising observation since microcompartments are energetically expensive structures. It has been assumed that swarmer cells minimize cellular metabolism, diverting cellular resources to motility (Armitage, 1981; Falkinham and Hoffman, 1984).

Phylogenetic analysis of *cut* genes reveals that they are predominately found in *Gammaproteobacteria* (particularly *Enterobacteriaceae*), *Firmicutes* and *Deltaproteobacteria* (Fig. 1). The *Gammaproteobacteria* contain similar, but phylogenetically distinct *cutC* genes from the other bacterial families. The CutC found in *Enterobacteriaceae*, such as *P. mirabilis*, is longer than those from obligate anaerobes, with an additional ∼300 amino acids towards the N‐terminus. The function of the additional N‐terminal portion of type II.a CutC is currently unknown. Because type II.a CutC was only identified from facultative anaerobes, we postulate that this extra N‐terminus of CutC may be involved in stabilizing the enzyme during transient oxygen exposure, therefore allowing them to thrive on intestinal or urinary tract epithelia.

Another possibility is that N‐terminus of CutC may be involved in the assembly of microcompartment. We have observed aberrant microcompartment structures in the cells of both the *cutC::kan* mutant and the complemented mutant (Fig. 5B), which resemble the swiss‐role like structures that are formed when only three Pdu shell proteins (PduA, PduB and PduJ) are recombinantly produced in *E. coli* (Parsons *et al*., 2010). One of the proteins, PduA, is a major component of the Pdu shell and has been shown to self‐assemble into sheets and higher order structures when produced in *E. coli* on its own (Pang *et al*., 2014). Therefore it is conceivable that, in the absence of CutC, multiple PduA‐like Cut proteins may assemble to form sheet‐like structures that appear to be layered and rolled up. This indicates that CutC may be necessary for correct assembly of the microcompartment, possibly by providing a structural scaffold on to which the shell is assembled. Clearly the role of CutC in microcompartments formation warrants further investigation.

In conclusion, the choline utilization mechanism in our model organism *P. mirabilis* represents an example of a human‐niche adaptation in *Enterobacteriaceae*, and further *in vivo* studies to test if this trait confers an advantage in the gut environment is certainly warranted. Metagenomic analysis of the gut microbiome has provided an important insight into the microbial communities in relation to gut health and disease, but understanding microbial metabolism helps to explain the ecology of the microbiota in our gut. Here, we have shown how choline is utilized as a carbon and energy source by *P. mirabilis* to facilitate growth and swarming. We have also identified a novel microcompartment involved in choline metabolism in this bacterium. Our observations represent a mechanism to help to understand better the interplay between the host and the gut microbiota and may contribute to explain the success of these bacteria during gut dysbiosis in future *in vivo* studies.

## Experimental procedures

### Bacterial strains and culture conditions

*Proteus mirabilis* DSM4479 (type‐strain, obtained from Deutsche Sammlung von Mikroorganismen und Zellkulturen GmbH, Braunschweig, Germany) was used in this study. *Escherichia coli* JM109 (Sigma‐Aldrich Company, Dorset) was used for cloning, and *E. coli* BLR(DE3)pLysS (Novagen, Merck, Darmstadt, Germany) for protein expression.

A defined medium was used for *P. mirabilis* to compare its growth on various carbon sources (Postgate and Kent, 1985), which contained per litre: 7 g K_2_HPO_4_, 3 g KH_2_PO_4_, 0.1 g MgSO_4_●7H_2_O, 1 g (NH_4_)_2_SO_4_ supplemented with: 5 ml micronutrients (per litre: 3 g each L‐histidine, L‐tryptophan, nicotinamide and L‐isoleucine), 50 μM ferric citrate, 50 mM sodium fumarate and either 10 mM glucose, glycerol or choline. For isolation of antibiotic‐cassette‐containing *P. mirabilis*, chloramphenicol (200 μg ml^−1^), ampicillin (150 μg ml^−1^) or kanamycin (150 μg ml^−1^) was added to growth medium as required. Anaerobic broth cultures of *P. mirabilis* were grown in 90 ml of medium, in crimp‐sealed 125 ml serum vials, at 37°C without shaking. Starter cultures were inoculated from a single colony and grown for 1–2 days at 37°C, without shaking, until OD_600_ reaches ∼ 0.6. To establish fully anaerobic conditions, serum vials were de‐gassed for 30 min with O_2_‐free N_2_ gas. The optical density at 600 nm (OD_600_) was measured every 2–24 h dependent on growth rate to determine culture density. Anaerobic swarming agar consisted of the defined medium described above, supplemented with yeast extract (0.025%, w/v) and solidified with 1.5% (w/v) agarose. *Proteus mirabilis* swarm plates were cultured at 30°C in an anaerobic chamber (Don Whitley Scientific, Shipley, UK).

The growth of *E. coli* expressing *P. mirabilis* proteins was carried out anaerobically in a modified M9 medium supplemented with 40 mM NaNO_3_, 400 mM NaCl, 50 μM FeCl_3_, 30 mM glucose and yeast extract (0.05%, w/v). A high concentration of salt (400 mM) was used to increase choline uptake by *E. coli* through increased expression of the choline transporter *betT* (Lamark *et al*., 1991). Wild‐type strains and mutant construction was carried out aerobically at 37°C in liquid Luria–Bertani (LB) broth or LB agar plates for *E. coli* strains and non‐swarming LB agar plates (per litre: 10 g tryptone, 5 g yeast extract, 0.5 g NaCl, 5 ml glycerol, solidified with 1.5% agar, w/v) for *P. mirabilis*.

### Phylogenetic analysis of choline TMA‐lyase genes

The CutC sequence of *Desulfovibrio desulfuricans* (Craciun and Balskus, 2012) was used to search the National Center for Biotechnology Information and Joint Genome Institute‐Integrated Microbial Genomes (JGI‐IMG) (http://img.jgi.doe.gov/) databases for its homologues using the blastp algorithm. The resultant sequences from this analysis were used to construct a phylogenetic tree in the ARB database (Ludwig *et al*., 2004). The neighbour‐joining method was used to infer the phylogeny of amino acid sequences of CutC and other enzymes of the glycyl radical enzyme family (Lehtiö and Goldman, 2004).

### Heterologous expression of *P* *. mirabilis* choline‐TMA lyase *CutC* and *CutD*

*cutC* (PMI2716) and *cutD* (PMI2715) of *P. mirabilis* were codon optimized for heterologous expression in *E. coli* (GenScript, Piscatsaway, USA). The codon‐optimized *cutC* was ligated into multiple cloning site (MCS) 1 and *cutD* into MCS2 of the expression vector pCOLADuet‐1 (Novagen, Merck, Darmstadt, Germany) to give the vector pCOLADuet‐CutC/D. A second construct was made in which the glycine residue (G1126) that provides the glycyl radical of CutC was replaced with alanine to give the vector pCOLADuet‐CutCA/D. These vectors were transformed into the expression strain *E. coli* BLR(DE3)pLysS. Expression of the proteins was induced by the addition of IPTG to a final concentration of 2 mM. The expression and identity of recombinant CutC and CutD were confirmed by SDS – polyacrylamide gel electrophoresis (SDS‐PAGE) combined with MALDI‐TOF (Fig. S2).

### Construction and complementation of c*ut* *C* mutant in *P* *. mirabilis*

Electrocompetent *P. mirabilis* cells were made following a method adapted from Visalli and colleagues (2003). Cells were grown aerobically in LB broth without NaCl at 37°C for 2.5–4 h. Mid‐log phase *P. mirabilis* cultures were harvested when they reached OD_600_ of 0.3–0.6. Before electroporation, thawed competent cells were combined with 500 ng plasmid deoxyribonucleic acid (DNA) and incubated on ice for 1 h. *Proteus mirabilis* was electroporated in a 1 mm gap cuvette at 1.8 kV, and the time constant was 4.5–4.6 ms. A kanamycin‐resistance gene was inserted into the *cutC* gene using the TargeTron targeted transposon mutagenesis system (Sigma‐Aldrich, Dorset) following the manufacturer's instructions. Briefly, a group II intron was reprogrammed to insert into the c*utC* gene by mutagenic polymerase chain reaction (PCR), using the following primers.

1 IBS 5′‐AAAAAAGCTTATAATTATCCTTAAAATTCCATCTGGTGCGCCCAGATAGGGTG‐3′
2 EBS2 5′‐TGAACGCAAGTTTCTAATTTCGGTTAATTTCCGATAGAGGAAAGTGTCT‐3′
3 EBS1d 5′‐CAGATTGTACAAATGTGGTGATAACAGATAAGTCCATCTGACTAACTTACCTTTCTTTGT‐3′

The re‐targeted intron was cloned into pGEM‐T and confirmed by DNA sequencing. The re‐targeted intron was then ligated into the plasmid pACD4K‐C to create pACD4K‐CutC. To create the *cutC* mutant, *P. mirabilis* competent cells were electroporated with the helper plasmid pAR1219 (Davanloo *et al*., 1984). Successful transformants were selected on non‐swarming agar plates containing ampicillin (150 μg ml^−1^). Electrocompetent *P. mirabilis* [pAR1219] were prepared as described above which were electroporated with the plasmid pACD4K‐CutC. Cells containing pACD4K‐CutC were selected for by overnight growth in LB broth containing ampicillin (150 μg ml^−1^), chloramphenicol (200 μg ml^−1^) and 1% (w/v) glucose. The addition of 0.5 mM IPTG to the chloramphenicol and ampicillin‐resistant isolate induced the intron to insert into the *cutC* gene in the *P. mirabilis* chromosome to create the *cutC::kan* mutant. Successful transformants were selected on non‐swarming agar plates containing kanamycin (150 μg ml^−1^). Curing for the helper plasmid pAR1219 in the transformants was carried out by growing on non‐swarming agar plates and selected with ampicillin (150 μg ml^−1^). To complement the *cutC::kan* mutant, the native *cutCD* (PMI2716/15) of *P. mirabilis* was amplified via PCR and cloned into pGEM‐T. The PCR primers used are cutC‐F (5′‐TCAGATGGGATCAGCCCTAC‐3′) and cutD‐R (5′‐CCTCCAAATCTGCATCAAAC‐3′). The *lacZ* promoter located in pGEM‐T was used to express *cutCD*, the orientation of which was confirmed by DNA sequencing. The pGEM‐T‐*cutCD* vector was electroporated into the *P. mirabilis cutC::kan* mutant and selected on non‐swarming agar plates containing ampicillin (150 μg ml^−1^).

### Growth of *P* *. mirabilis* on choline and quantification for choline and TMA

*Proteus mirabilis* cells were grown anaerobically at 37°C in the aforementioned defined medium. The growth rate of *P. mirabilis* strains was compared in the defined medium when either, 10 mM glucose, glycerol or choline were used as the sole carbon source, or with 10 mM of either glucose or glycerol supplemental with 10 mM choline. The concentrations of TMA and choline in the media were determined using ion chromatography on an 881 Compact IC Pro (Metrohm, Herisau, Switzerland) as described previously (Zhu *et al*., 2014).

### *P* *. mirabilis* swarming assays

Anaerobic swarming was performed using the defined medium as described above to test the effect of choline on swarming interval and colony expansion rate. Plates were inoculated anaerobically with 2 μl *P. mirabilis* inoculum culture. The *P. mirabilis* inoculum consisted of an overnight anaerobic culture grown in the defined medium to an OD_600_ of 0.4–0.6. Inoculated anaerobic swarm plates were incubated at 30°C in an anaerobic growth cabinet (Don Whitley Scientific, Shipley, UK). Swarm radius was measured on triplicate plates with glucose or glycerol supplemented with choline. The radius of the total growth on anaerobic swarming agar plates was measured from the central point of inoculation in four directions at 90° to each other every 8–16 h.

### Ribonucleic acid isolation and transcriptome analysis by RNA‐Seq

Five replicate *P. mirabilis* cultures were grown anaerobically in the defined medium with glucose only or glucose plus choline. Cells were harvested by centrifugation at 4000 rpm for 20 min when the OD_600_ reached 0.4. Total ribonucleic acid (RNA) was extracted using the TRI reagent (Sigma‐Aldrich, Dorset). TRI reagent and 1‐bromo‐3‐chloropropane were mixed with the cell pellet and transferred to MaXtract High Density columns (Qiagen, Crawley, UK). Phase separation of the RNA aqueous layer from DNA and proteins was carried out following the manufacturer's protocols. The resultant aqueous layer was treated with on‐column DNAse (Qiagen, Crawley, UK) and purified using an RNeasy Mini Kit (Qiagen, Crawley, UK). Purified RNA was re‐suspended in 60 μl RNase‐free water. The Ribo‐Zero rRNA Removal Kit (Gram‐Negative Bacteria, Epicentre, Cambio, UK) was used to enrich messenger RNA. Ribonucleic acid quality and quantity was assessed on an Agilent bioanalyser (Agilent, Edinburgh, UK). Complementary DNA Libraries were prepared using ScriptSeq v2 RNA‐Seq Library Preparation Kit (Epicentre, Cambio, UK). One hundred bp paired‐end sequencing was carried out on an Illumina HiSeq 2500 platform (Illumina, Little Chesterfield, UK). The raw Illumina RNA‐seq reads were trimmed and filtered using the FASTX Toolkit to ensure only high‐quality sequence data was retained. The Tophat program was used to align the RNA‐seq reads to the *P. mirabilis* HI4320 genome sequence. Read counts were extracted using the HTSeq Python package. The r software package DEseq was used to determine which genes were differentially expressed between the cultures grown with and without choline (Anders and Huber, 2010). Count data were normalized by the size factor for each sample to make the count values comparable. The *P* value cut‐off was set at *P*‐value < 0.05.

### TEM imaging of *P* *. mirabilis* microcompartments

Anaerobic liquid cultures of *P. mirabilis* were grown in 80 ml defined medium supplemented with glucose, choline or glucose plus choline. Liquid cultures of *P. mirabilis* were fixed for 14 h by injection with fixative to a final concentration of 2.5% (w/v) glutaraldehyde in 100 mM sodium cacodylate (pH 7.2). The cells were harvested by centrifugation at 4000 r.p.m. for 15 min and approximately half the cell pellet was used, which was washed twice in 2 ml 100 mM sodium cacodylate (pH 7.2). Cells were post‐fixed for 2 h in 1% (w/v) osmium tetroxide, followed by three washes with the sodium cacodylate buffer before dehydration. Dehydration: 50% (v/v) ethanol for 15 min, 70% ethanol overnight, 90% ethanol for 15 min, then three times 100% dry ethanol for 15 min and two washes with propylene oxide for 15 min. Embedding in agar low viscosity (LV) was carried out as follows: propylene oxide: LV resin at 1:1 for 30 min followed by incubation 2 × 1.5 h in 100% freshly made agar LV resin. The samples were placed in 0.5 ml BEEM capsules, centrifuged for 5 min at 900 × g to concentrate the cells to the tip and incubated at 60°C for 24 h to polymerize.

Agar wedges were cut from anaerobic *P. mirabilis* swarming culture plates. The agar wedges were briefly immersed in 0.1% alcian blue (w/v) in 0.1% acetic acid (v/v) to stain the agar for easy handling of the samples for TEM processing as described above. The processed wedges were then placed in aluminium dish moulds with *P. mirabilis* swarming culture facing down in LV resin and incubated at 60°C for 24 h to polymerize.

All specimens were thin sectioned (65 nm) with a diamond knife on an RMC MT‐6000‐XL ultramicrotome. Sections were collected on 400 mesh copper grids, post‐stained with 4.5% (w/v) uranyl acetate in 1% acetic acid (v/v) for 45 min and Reynolds lead citrate for 7 min at room temperature. Sections were then observed on a Jeol 1230 transmission electron microscope operated at an accelerating voltage of 80 kV. Images were captured on a Gatan MultiScan 791 digital camera.

## Supporting information


**Fig. S1.** Alignment of selected CutC amino acid sequences. The top four protein sequences represent the *P. mirabilis‐*like type II cluster, the first three of which are type II.a *Gammaproteobacteria* and the *cut* cluster contains the GRM2 class of microcompartment proteins, while the fourth sequence, *D. reducens* is a type II.b *Firmicute* and contains the GRM1 class of microcompartment proteins. The lower four sequences represent the *D. desulfuricans‐*like type I cluster and all contain the GRM1 class of microcompartment proteins. The type II.a cluster have ∼ 300 extra amino acids at the N terminus. Amino acid positions with black or grey background shading indicate poor conservation (0–70%). The blue closed circle and blue box indicate the position of the crucial conserved glycine residue that forms the glycyl radical.
**Fig. S2.** SDS‐PAGE analyses of cell lysate from *E. coli* overexpressing codon‐optimized *P. mirabilis* CutC and CutD (A) supernatant and (B) pellet. Lanes 1–3 CutCD induced with IPTG; 4–6. CutC(G1126A)/CutD induced with IPTG; 7–8 un‐induced control. Arrows indicate the presence of 127 kDa CutC (A) and 36 kDa CutD (B) in lanes 1–6 respectively.
**Fig. S3.** Cumulative anaerobic swarm‐colony radiuses of *P. mirabilis* incubated at 30°C, inoculated from an anaerobic broth culture. Error bars show standard deviation for three replicate plates.A. The carbon sources on the swarming agar plates are choline (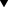), glycerol only (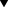) or varying concentrations of glycerol and choline (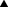).B. The carbon sources are glycerol only (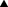) or varying concentrations of glycerol and TMA (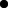). Maximum swarm radius is 42 mm on petri dishes.
**Fig. S4.** Anaerobic growth of *P. mirabilis* in liquid broth cultures in a defined medium at 37°C. (A) wild‐type; (B) *cutC::kan* mutant and (C) *cutC::kan* mutant complemented with native *cutCD*.
**Fig. S5.** The *P. mirabilis* genetic *cut* island and the proposed enzyme functions of the component *cut* genes. The prediction of promoter sites was conducted using three web‐based programs. bprom (http://linux1.softberry.com/berry.phtml), PePPER (http://pepper.molgenrug.nl/) and bdgp (http://www.fruitfly.org/seq_tools/promoter.html). The predicted promoter sites shown in the figure represent the top predicted sites on the coding strand, predicted by at least two of the programs.
**Fig. S6.** Transmission electron microscopy micrographs of *P. mirabilis* cultured in liquid minimal medium at early time points. The medium was supplemented with glucose, choline or glucose plus choline. Cells were harvested after 4 h, 6 h and 8 h growth in the minimal medium. Microcompartments are indicated by red arrows. EPS, extracellular polysaccharides.
**Fig. S7.** Time course from which TEM images in Fig. S4 were compiled showing choline degradation and trimethylamine production, overlaid with growth curves, for anaerobically grown broth cultures of *P. mirabilis* grown on: A. glucose; B. choline and C. glucose plus choline.
**Fig. S8.** The *cut* promoter in induced by choline. The promoter of the *cut* gene cluster (∼580 bp upstream of PMI2722) was cloned into the promoterless *lacZ*‐probe vector pBIO1878 (Todd *et al*., 2012). The resulting plasmid was electroporated into wild‐type *P. mirabilis* and selected on spectinomycin (150 μg ml^−1^). The transformant was cultivated in the defined liquid medium supplemented with either glucose or choline as the sole carbon source and the activity of LacZ was quantified after 40 h growth.
