# Supplementary material for: Anaerobic choline metabolism in microcompartments promotes growth and swarming of P roteus mirabilis
Source: Environ Microbiol. 2015 Nov 3;18(9):2886–98. doi: 10.1111/1462-2920.13059 (PMC5026066; doi:10.1111/1462-2920.13059)
Supplement: Supplementary file 1 — Fig. S1. Alignment of selected CutC amino acid sequences. The top four protein sequences represent the P. mirabilis‐like type II cluster, the first three of which are type II.a Gammaproteobacteria and the cut cluster contains the GRM2 class of microcompartment proteins, while the fourth sequence, D. reducens is a type II.b Firmicute and contains the GRM1 class of microcompartment proteins. The lower four sequences represent the D. desulfuricans‐like type I cluster and all contain the GRM1 class of microcompartment proteins. The type II.a cluster have ∼ 300 extra amino acids at the N terminus. Amino acid positions with black or grey background shading indicate poor conservation (0–70%). The blue closed circle and blue box indicate the position of the crucial conserved glycine residue that forms the glycyl radical. Fig. S2. SDS‐PAGE analyses of cell lysate from E. coli overexpressing codon‐optimized P. mirabilis CutC and CutD (A) supernatant and (B) pellet. Lanes 1–3 CutCD induced with IPTG; 4–6. CutC(G1126A)/CutD induced with IPTG; 7–8 un‐induced control. Arrows indicate the presence of 127 kDa CutC (A) and 36 kDa CutD (B) in lanes 1–6 respectively. Fig. S3. Cumulative anaerobic swarm‐colony radiuses of P. mirabilis incubated at 30°C, inoculated from an anaerobic broth culture. Error bars show standard deviation for three replicate plates. A. The carbon sources on the swarming agar plates are choline (), glycerol only () or varying concentrations of glycerol and choline (). B. The carbon sources are glycerol only () or varying concentrations of glycerol and TMA (). Maximum swarm radius is 42 mm on petri dishes. Fig. S4. Anaerobic growth of P. mirabilis in liquid broth cultures in a defined medium at 37°C. (A) wild‐type; (B) cutC::kan mutant and (C) cutC::kan mutant complemented with native cutCD. Fig. S5. The P. mirabilis genetic cut island and the proposed enzyme functions of the component cut genes. The prediction of promoter sites was conducted using three web‐bas [file EMI-18-2886-s001.zip › EMI_13059_supp-0001-revised supplementary information.docx]

Supplementary Figure 1. Alignment of selected CutC amino acid sequences. The top 4 protein sequences represent the *P. mirabilis-*like type II cluster, the first 3 of which are type II.a *Gammaproteobacteria* and the *cut* cluster contains the GRM2 class of microcompartment proteins, while the fourth sequence, *D. reducens* is a type II.b *Firmicute* and contains the GRM1 class of microcompartment proteins. The lower 4 sequences represent the *D. desulfuricans-*like type I cluster and all contain the GRM1 class of microcompartment proteins. The type II.a cluster have ~ 300 extra amino acids at the N terminus. Amino acid positions with black or grey background shading indicates poor conservation (0-70%). The blue closed circle and blue box indicate the position of the crucial conserved glycine residue that forms the glycyl radical.

Supplementary Figure 2. SDS-PAGE analyses of cell lysate from *E. coli* over-expressing codon-optimised *P. mirabilis* CutC and CutD A. supernatant and B. pellet. Lanes 1-3 CutCD induced with IPTG; 4-6. CutC(G1126A)/CutD induced with IPTG; 7-8 un-induced control. Arrows indicate the presence of 127 kDa CutC (A) and 36 kDa CutD (B) in lanes 1-6, respectively.

**Supplementary Figure 3.** Cumulative anaerobic swarm-colony radiuses of *P. mirabilis* incubated at 30 °C, inoculated from an anaerobic broth culture. Error bars show standard deviation for three replicate plates. **A.** The carbon sources on the swarming agar plates are choline (●), glycerol only (▲) or varying concentrations of glycerol and choline (◆). **B.** The carbon sources are glycerol only (▲) or varying concentrations of glycerol and TMA (●). Maximum swarm radius is 42 mm on petri dishes.

**Supplementary Figure 4.** Anaerobic growth of *P. mirabilis* in liquid broth cultures in a defined medium at 37 °C. **A**. wild-type; **B**. *cutC::kan* mutant and **C**. *cutC::kan* mutant complemented with native *cutCD*.

Supplementary Figure 5. The *P. mirabilis* genetic *cut* island and the proposed enzyme functions of the component *cut* genes. The prediction of promoter sites was conducted using three web-based programs. BPROM (http://linux1.softberry.com/berry.phtml), PePPER (http://pepper.molgenrug.nl/) and BDGP (http://www.fruitfly.org/seq_tools/promoter.html). The predicted promoter sites shown in the figure represent the top predicted sites on the coding strand, predicted by at least two of the programs.

Supplementary Figure 6. TEM micrographs of *P. mirabilis* cultured in liquid minimal medium at early time-points. The medium was supplemented with glucose, choline or glucose plus choline. Cells were harvested after 4, 6 and 8 hours growth in the minimal medium. Microcompartments are indicated by red arrows. EPS, extracellular polysaccharides.

Supplementary Figure 7. Time-course from which TEM images in Supplementary Figure 4 were compiled showing choline degradation and trimethylamine production, overlaid with growth curves, for anaerobically grown broth cultures of *P. mirabilis* grown on: A. glucose B. choline and C. glucose plus choline.

Supplementary Figure 8. The *cut* promoter in induced by choline. The promoter of the *cut* gene cluster (~580 bp upstream of PMI2722) was cloned into the promoterless *lacZ*-probe vector pBIO1878 (Todd et al., 2012). The resulting plasmid was electroporated into wild-type *P. mirabilis* and selected on spectinomycin (150 μg ml^-1^). The transformant was cultivated in the defined liquid medium supplemented with either glucose or choline as the sole carbon source and the activity of LacZ was quantified after 40 hours growth.

Reference

Todd JD, Kirkwood M, Newton-Payne S, Johnston AWB (2012) DddW, a third DMSP lyase in a model *Roseobacter* marine bacterium, *Ruegeria pomeroyi* DSS-3. ISME J 6(1):223–226.
