# Supplementary material for: Anaerobic choline metabolism in microcompartments promotes growth and swarming of P roteus mirabilis
Source: Environ Microbiol. 2015 Nov 3;18(9):2886–98. doi: 10.1111/1462-2920.13059 (PMC5026066; doi:10.1111/1462-2920.13059)
Supplement: Supplementary file 1 — Fig. S1. Alignment of selected CutC amino acid sequences. The top four protein sequences represent the P. mirabilis‐like type II cluster, the first three of which are type II.a Gammaproteobacteria and the cut cluster contains the GRM2 class of microcompartment proteins, while the fourth sequence, D. reducens is a type II.b Firmicute and contains the GRM1 class of microcompartment proteins. The lower four sequences represent the D. desulfuricans‐like type I cluster and all contain the GRM1 class of microcompartment proteins. The type II.a cluster have ∼ 300 extra amino acids at the N terminus. Amino acid positions with black or grey background shading indicate poor conservation (0–70%). The blue closed circle and blue box indicate the position of the crucial conserved glycine residue that forms the glycyl radical. Fig. S2. SDS‐PAGE analyses of cell lysate from E. coli overexpressing codon‐optimized P. mirabilis CutC and CutD (A) supernatant and (B) pellet. Lanes 1–3 CutCD induced with IPTG; 4–6. CutC(G1126A)/CutD induced with IPTG; 7–8 un‐induced control. Arrows indicate the presence of 127 kDa CutC (A) and 36 kDa CutD (B) in lanes 1–6 respectively. Fig. S3. Cumulative anaerobic swarm‐colony radiuses of P. mirabilis incubated at 30°C, inoculated from an anaerobic broth culture. Error bars show standard deviation for three replicate plates. A. The carbon sources on the swarming agar plates are choline (), glycerol only () or varying concentrations of glycerol and choline (). B. The carbon sources are glycerol only () or varying concentrations of glycerol and TMA (). Maximum swarm radius is 42 mm on petri dishes. Fig. S4. Anaerobic growth of P. mirabilis in liquid broth cultures in a defined medium at 37°C. (A) wild‐type; (B) cutC::kan mutant and (C) cutC::kan mutant complemented with native cutCD. Fig. S5. The P. mirabilis genetic cut island and the proposed enzyme functions of the component cut genes. The prediction of promoter sites was conducted using three web‐bas [file EMI-18-2886-s001.zip › EMI_13059_supp-0002-Supplementary_figures.pptx]

## Slide 1
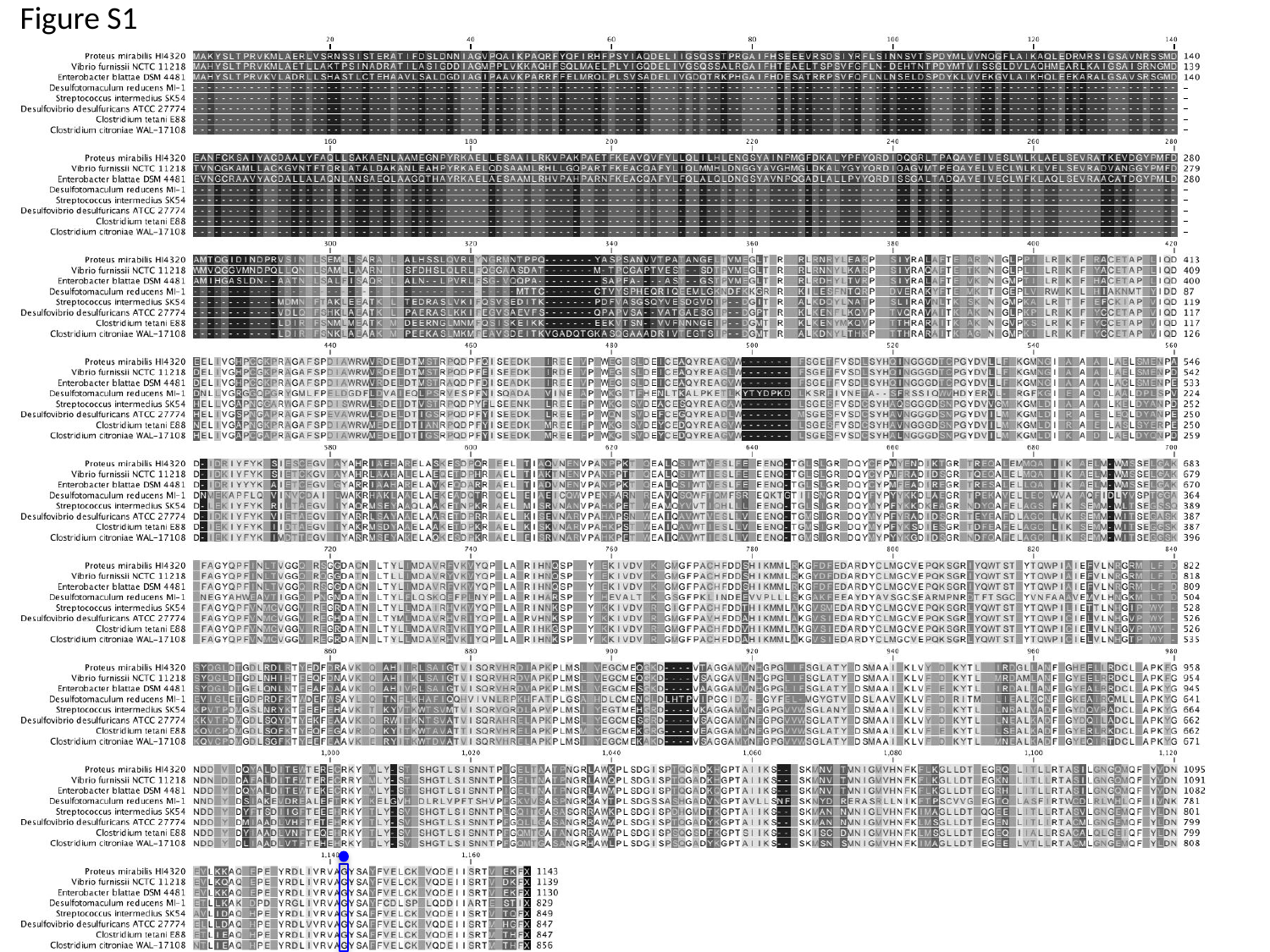

Figure S1

## Slide 2
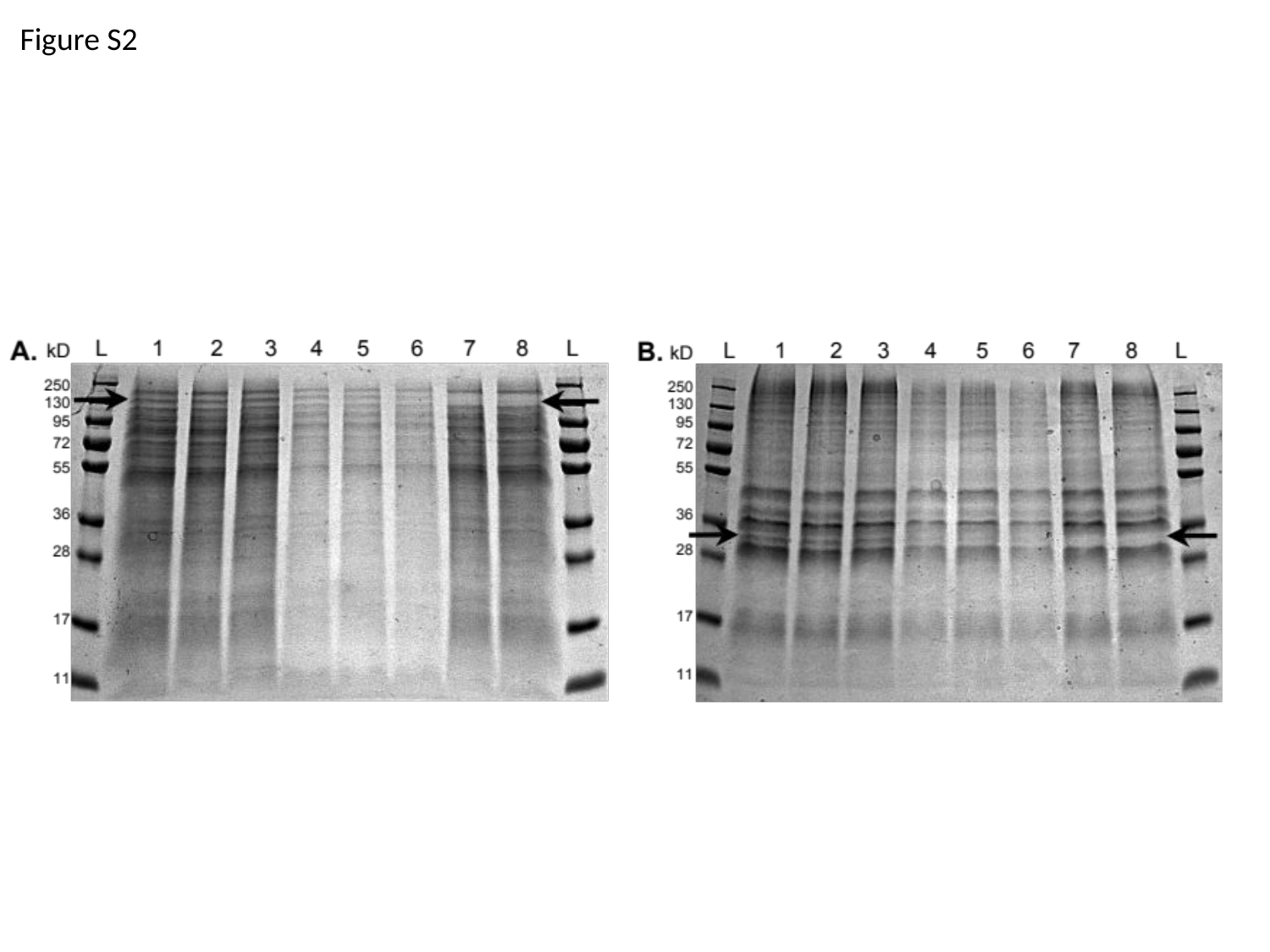

Figure S2

## Slide 3
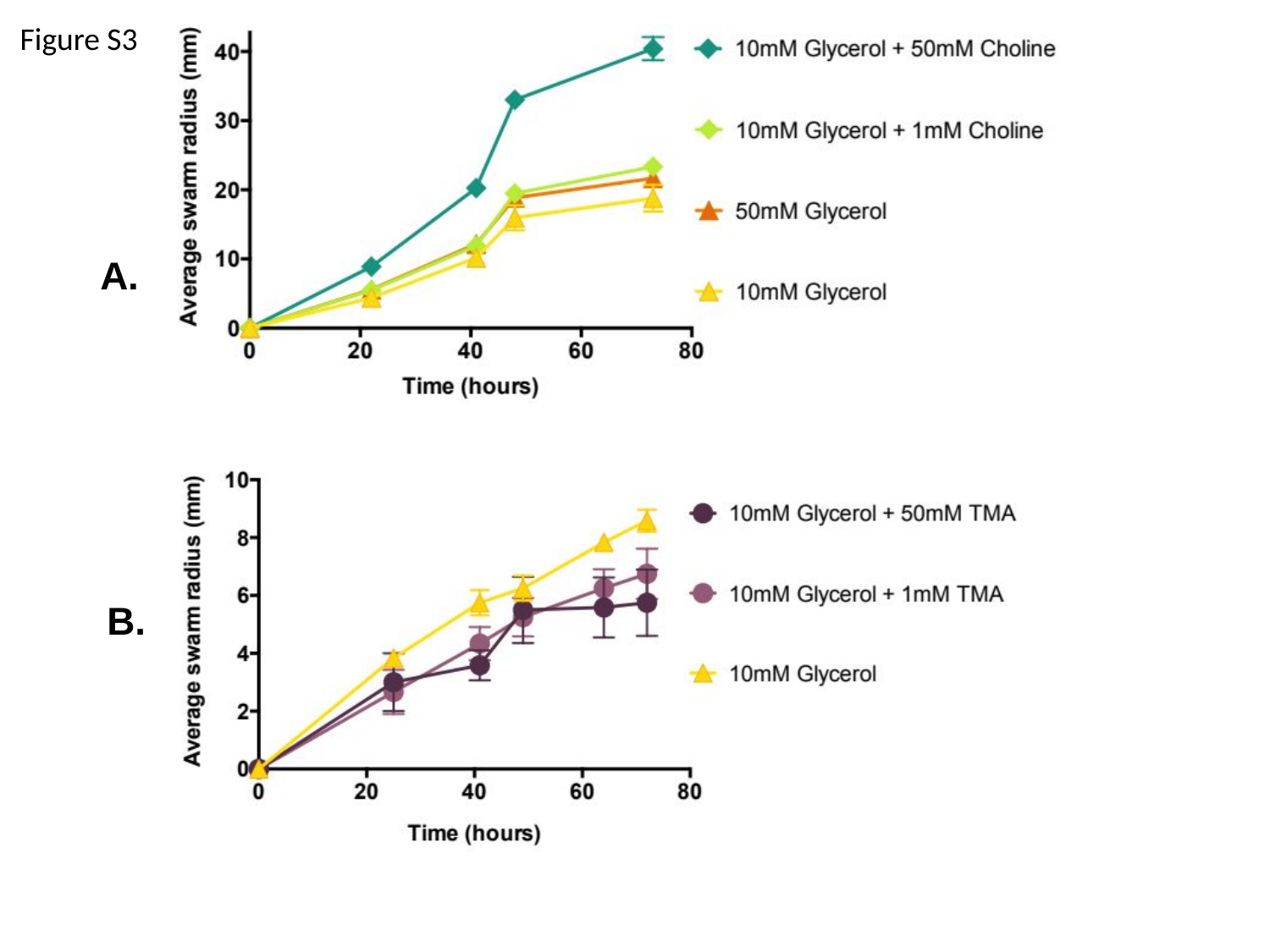

Figure S3
A.
B.

## Slide 4
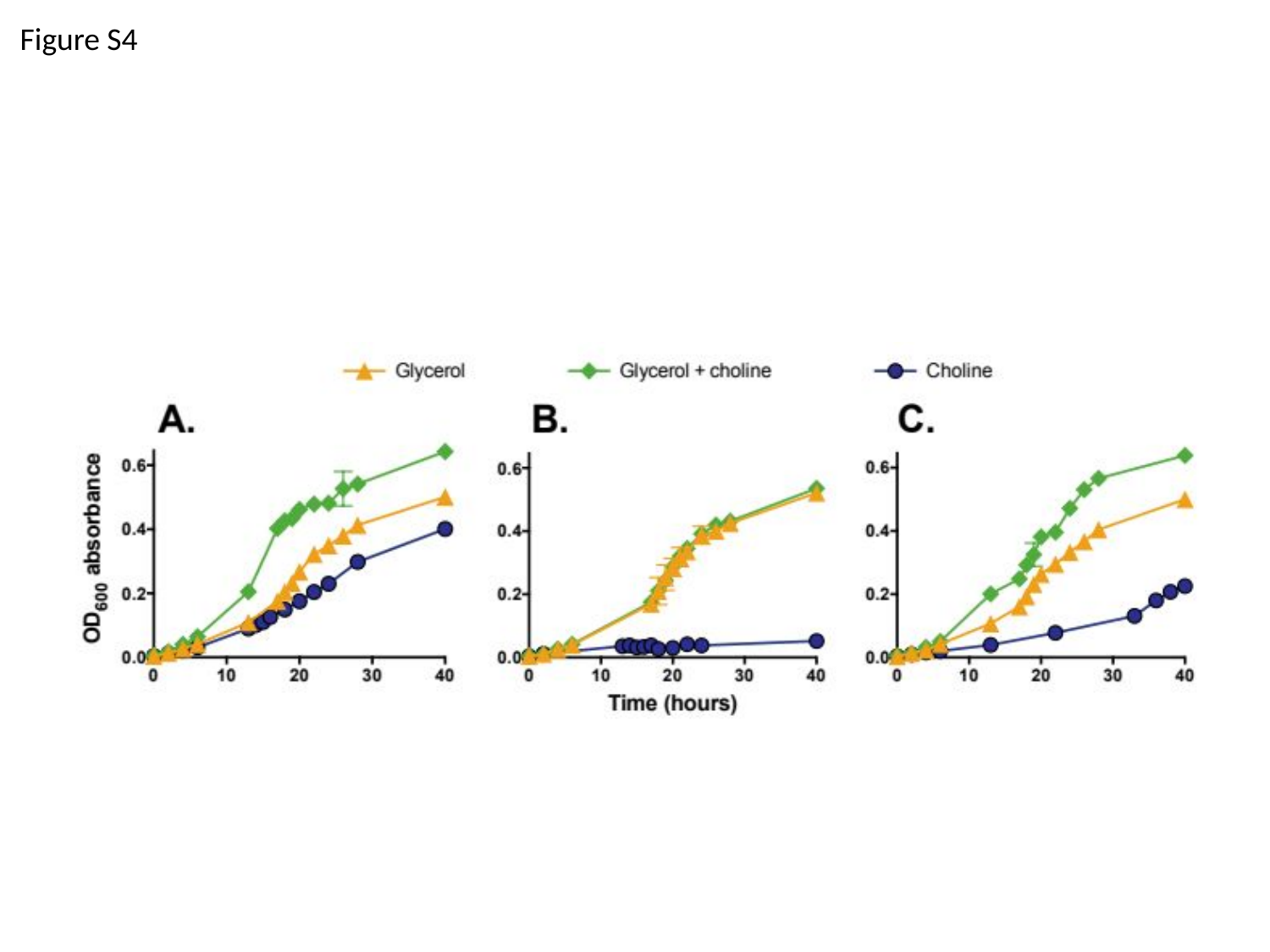

Figure S4

## Slide 5
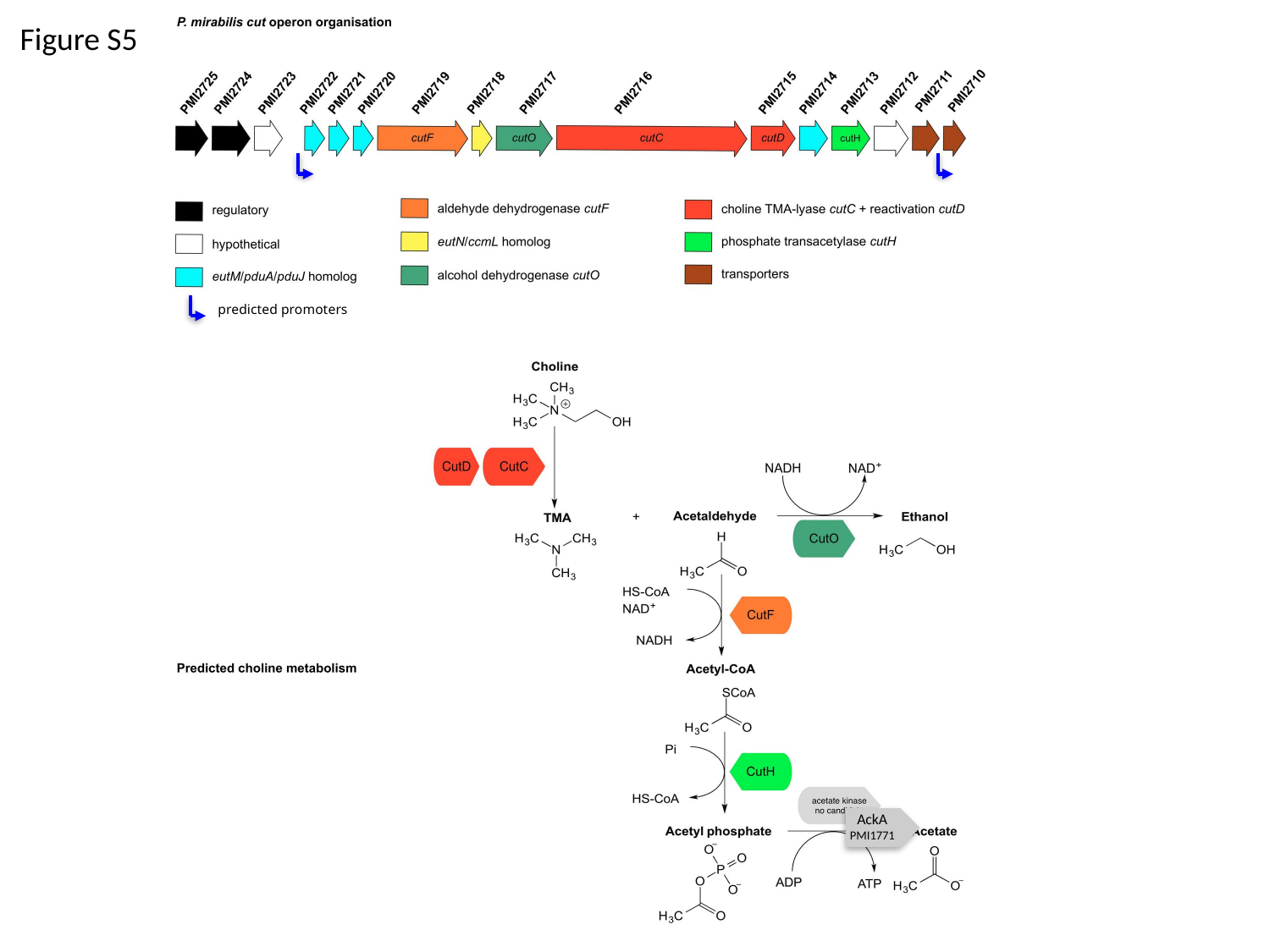

Figure S5
predicted promoters
AckA
PMI1771

## Slide 6
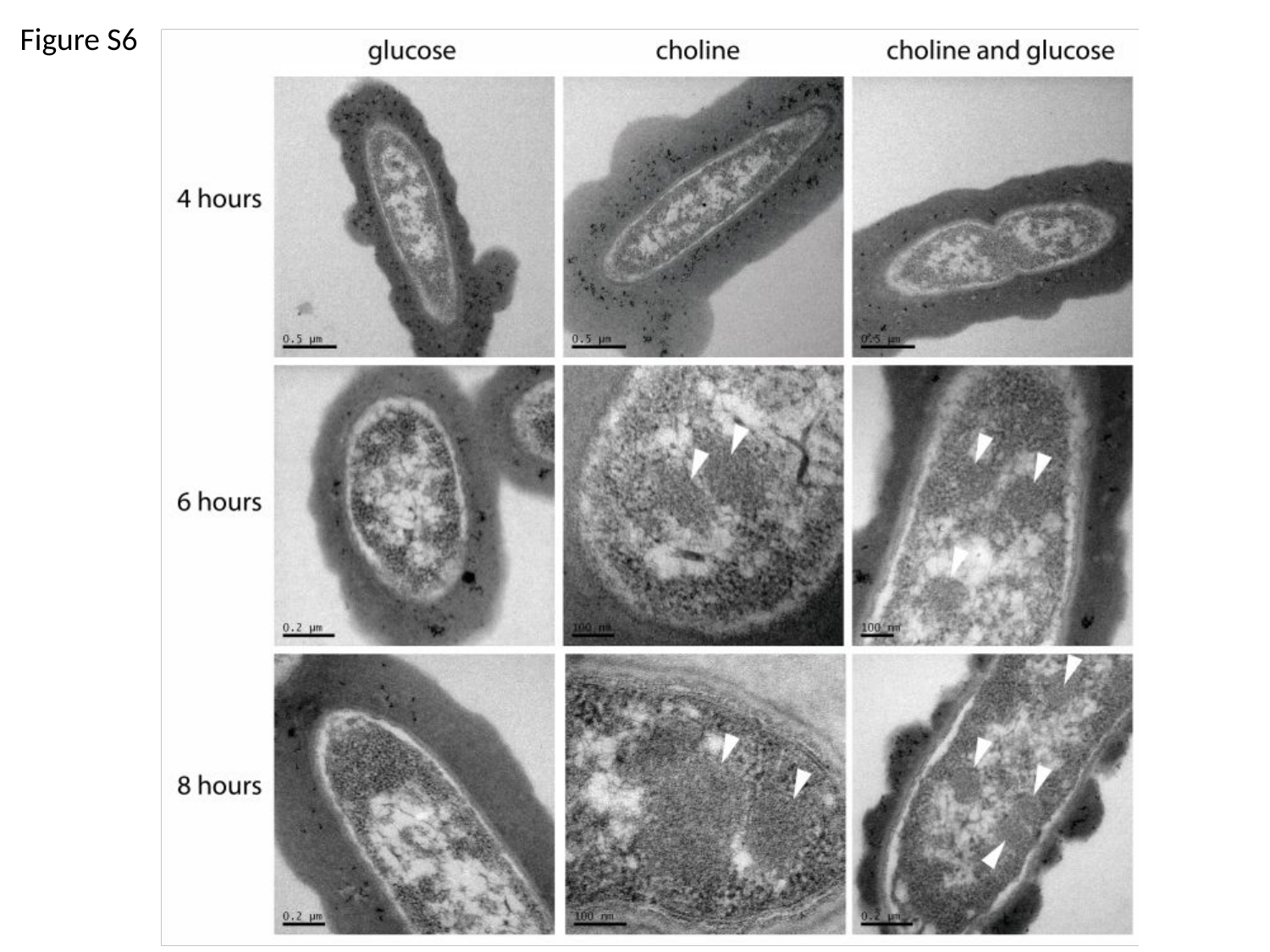

Figure S6

## Slide 7
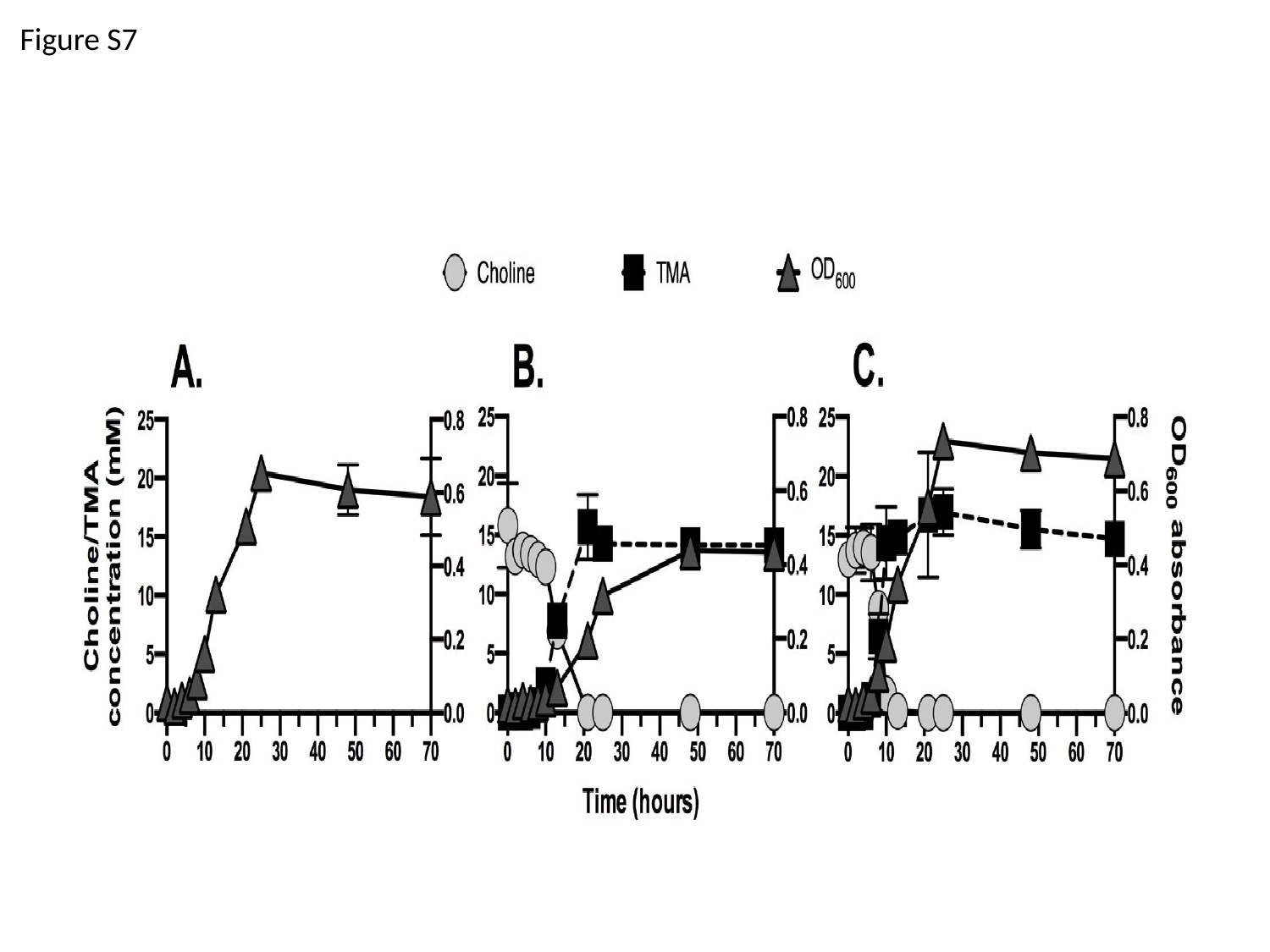

Figure S7

## Slide 8
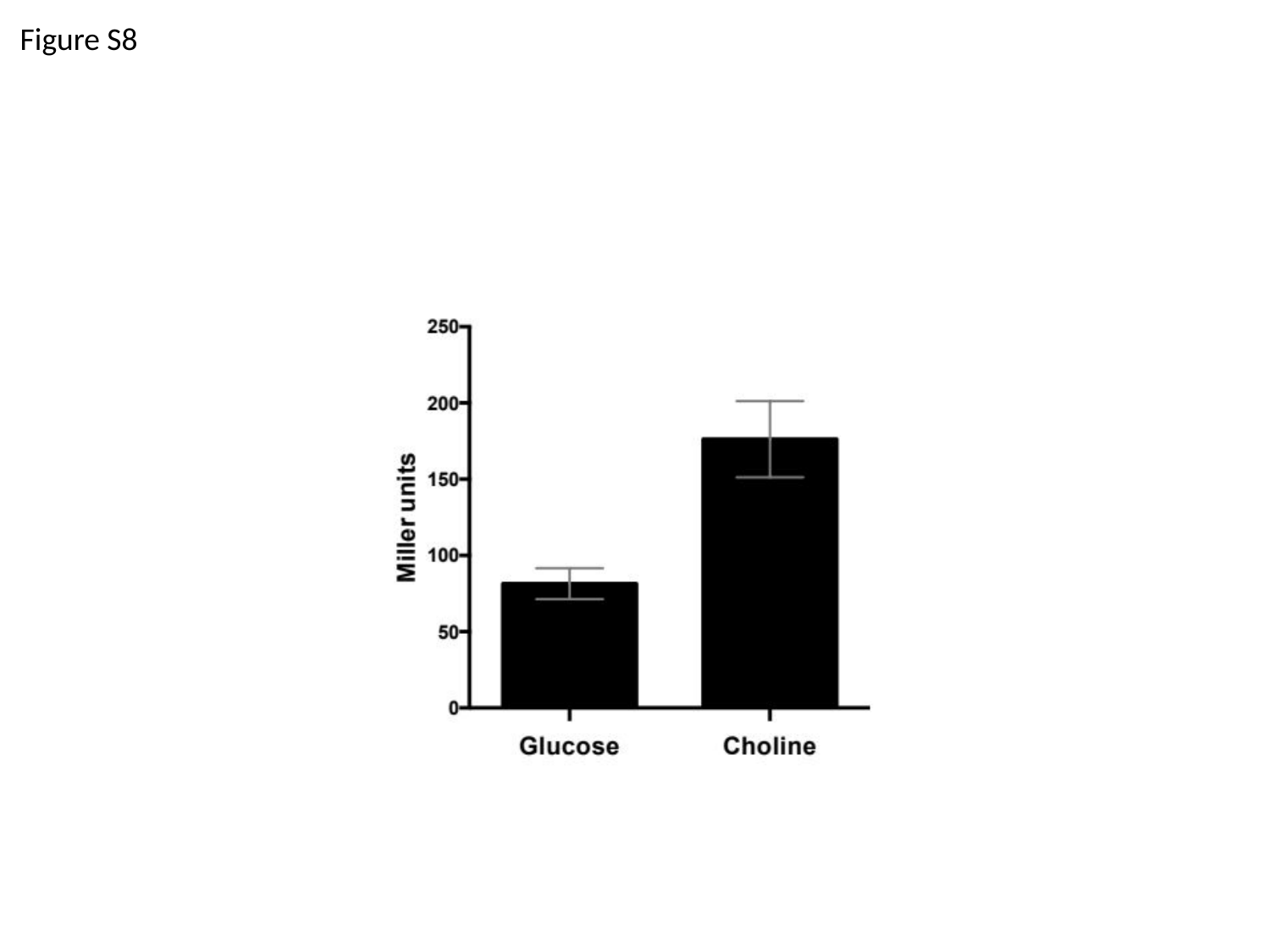

Figure S8
